# Diagnosis and Characters of Non-Specific Low Back Pain in Japan: The Yamaguchi Low Back Pain Study

**DOI:** 10.1371/journal.pone.0160454

**Published:** 2016-08-22

**Authors:** Hidenori Suzuki, Tsukasa Kanchiku, Yasuaki Imajo, Yuichiro Yoshida, Norihiro Nishida, Toshihiko Taguchi

**Affiliations:** Department of Orthopedics Surgery, Yamaguchi University Graduate School of Medicine, Yamaguchi, Japan; Tokai Daigaku, JAPAN

## Abstract

**Study Design:**

Cross sectional data from the Yamaguchi low back pain study conducted in Yamaguchi prefecture, Japan, was used for this analysis.

**Methods:**

A total of 320 patients were recruited from walk-in orthopedic clinics in Yamaguchi Prefecture, Japan. Patients visited the clinics primarily for low back pain (LBP) and sought treatment between April and May 2015. A self-questionnaire was completed by patients, while radiographic testing and neurological and physical examination was performed by the orthopedist in each hospital. The cause and characters of LBP was determined following examination of the data, regional anesthesia and block injection.

**Results:**

‘Specific LBP’ was diagnosed in 250 (78%) patients and non-diagnosable, ‘non-specific LBP’ in 70 (22%) patients. The VAS scores of patients were: LBP, 5.8±0.18; leg pain, 2.9±0.18 and the intensity of leg numbness was 1.9±0.16. Item scores for SF-8 were: general health, 46.6±0.40; physical function, 43.5±0.51; physical limitations, 42.8±0.53; body pain, 42.1±0.52; vitality, 48.4±0.37; social function, 46.9±0.53; emotional problems, 48.9±0.43; mental health, 46.9±0.43.

**Conclusions:**

The incidence of non-specific LBP in Japan was lower than previous reports from western countries, presumably because of variation in the diagnosis of LBP between different health care systems. In Japan, 78% of cases were classified as ‘specific LBP’ by orthopedists. Identification of the definitive cause of LBP should help to improve the quality of LBP treatment.

## Introduction

About one quarter of adults in Japan suffer from low back pain (LBP) [1]. Worldwide, the cause of LBP is unclear in about 85% of patients and such cases are referred to as ‘non-specific LBP’ [2,3]. LBP in which the cause cannot be clearly identified are also classified as ‘unknown origin of LBP’ and ‘unable to diagnose’. However careful examination by specialists can lead to more patients being diagnosed with a clear and treatable cause of LBP. Increasing the proportion of LBP cases with a clear diagnosis is important clinically because it allows proper treatment to begin early and this is the key factor for achieving improved outcome [4–7]. Earlier papers from North America reported on LBP diagnosis by primary care doctors at walk-in clinics and emergency hospitals [2,3]. These papers reported the proportion of diagnosable LBP with a clear cause was only 15–20% of patients with LBP. Japanese orthopedic doctors have felt the proportion of diagnosable LBP in these previous studies was quite low, despite accurate LBP diagnosis being the most important step prior to the start of treatment. However, the diagnosis and treatment of LBP is quite different in Japan, due partly to a different health insurance system. From the first contact, Japanese patients with LBP visit orthopedic clinics and are seen by specialist doctors.

In this study we investigated the diagnosis of LBP by orthopedists working in the Japanese clinical setting. We hypothesized that the incidence of an unclear diagnosis of LBP in this setting would be lower than in previous North American reports. We also hypothesized the lower percentage of diagnosable LBP in North America was due to initial examination by clinicians who were not specialized in the neurological and physical examinations required for accurate diagnosis of LBP. This was because of restricted access to medical specialists at first contact due to the North American health insurance system. We examined patients with LBP who visited orthopedic clinics during April and May of 2015 in Yamaguchi prefecture, Japan. These clinics belong to the Yamaguchi Clinical Orthopedics Association (YCOA). Our aim was to establish the reasons for the relatively higher percentage of diagnosable LBP in Japan, which could lead to better clinical results for these patients.

## Materials and Methods

This study was approved by the Institutional Review Board (IRB) of Yamaguchi University prior to the start of data collection. The participants provided written informed consent to participate in this study. Participant consent was recorded and the IRB of Yamaguchi University approved this consent procedure.

### Patients and data collection

All patients (age range, 20–85 years; average age, 55.7 years) sought treatment between April and May 2015 in the walk-in orthopedic clinics of private hospitals located in Yamaguchi Prefecture, Japan. The participant recruitment date was between April 15 2015 and May 31 2015. The inclusion criteria in this study was the patients from 20 to 85 years with LBP which was defined as experiencing pain, discomfort and stiffness in the lower back from the 12th rib to the lumbar or lumbosacral area, including lower limb symptoms. A total of 323 patients (160 men and 163 women) of whom we could obtain written consent to participate in the study were recruited during these visits to the hospital. Finally we used 320 patient’s data for this study because the data of 3 patients had some data missing. The clinics are a site of primary care for patients with bone, joint and nerve disorders. Patients who had financial issues relating to worker’s compensation or had a mental disorder and were not able to answer the questionnaire were excluded. Japanese patients with LBP usually attend these orthopedic clinics first to receive treatment and thus all patients are self-referred. In each hospital the patient is examined by an orthopedist.

A self-questionnaire was completed by patients, while radiographic testing and neurological and physical examinations were performed by the orthopedist in each hospital. The visual analog scales (VAS) with scores from 0 to 10 (0 = no pain, 10 = maximum pain) were used to measure the intensity of LBP and pain. In addition a score of 0 to 10 was used to quantify the intensity of lower limb numbness. The self-administered questionnaire designed for epidemiological surveys was used to obtain information including the occurrence of LBP, the duration of LBP and the length of absence from work. Self-questionnaires also included the Japanese Orthopedic Association Back Pain Evaluation Questionnaire (JOABPEC) [8] and SF-8 [9]. The treating doctor verified the Japanese Orthopedic Association score (JOA score) (Table 1) [10].

**Table 1:** JOA score.

1. Subjective symptoms (9 points)
a. Low back pain: None (3), occasional mild pain (2), frequent mild or occasional severe pain (1), frequent or continuous severe pain (0)
b. Leg pain and/or tingling: None (3), occasional slight symptom (2), frequent slight or occasional severe symptom (1), frequent or continuous severe symptom (0)
c. Walking capacity: Normal (3), Able to walk further than 500 metres although it results in pain, tingling and /or muscle weakness (2) Unable to walk further than 500 metres owing to leg pain, tingling and/or muscle weakness (1), Unable to walk further than 100 metres owing to leg pain, tingling and/or muscle weakness (0)
2. Objective findings (6 points)
a. SLR tests: Normal (2), 30° to 70° (1), < 30° (0)
b. Sensory disturbance: None (2), slight disturbance (1), marked disturbance (0)
c. Motor disturbance: Normal (grade 5) (2), slight weakness (grade 4) (1), marked weakness (grade 3) (0)
3. Restriction of ADL (14 points)
Turn over while lying, standing, washing the face, leaning forwards, sitting (about one hour), lifting or holding heavy objects, walking: No restriction (2), moderate restriction (1), severe restriction (0) for each item
4. Bladder function (-6 points)
Normal (0), mild dysuria (-3), severe dysuria (-6)

**Fig 1:**
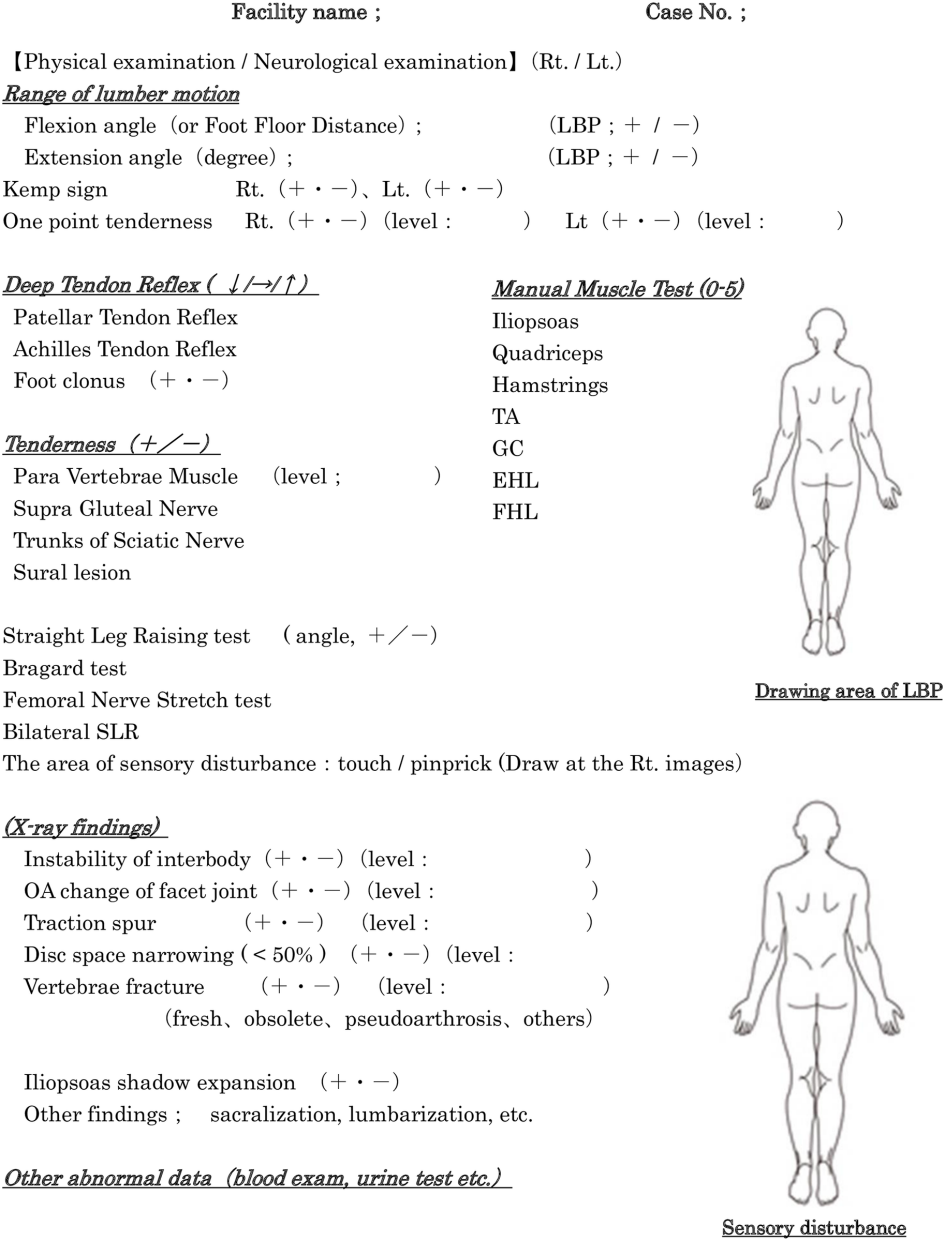
Common tools used for radiographic, neurological and physical examination. The orthopedist recorded all examination data for each LBP patient.

Common tools (Fig 1) were used for radiographic, neurological and physical examinations and the orthopedist recorded all examination data for each LBP patient. Following evaluation of this data, regional anesthesia and block injection, a final diagnosis was made for the cause of LBP (Table 2). For the definitive diagnosis of LBP caused by discogenic, facet joint, sacroiliac joint and lumbar disc herniation, the block injection of disc, facet and sacroiliac block was performed twice in all cases with 1 ml of 1% lidocaine and 0.5% bupivacaine to avoid the placebo effect. The limitation of this study was that we analyzed only the LBP patients who visited the orthopedics clinics in Yamaguchi prefecture in part of Japan and we collected the data of the LBP patients during the limited dates from April 15 2015 to May 31 2015. We didn’t revealed the results of treatment following LBP diagnosis in this study.

**Table 2:** Diagnosis of Low Back Pain.

1) Fascial lumbago
2) Facet joint syndrome
3) Discogenic lumbago
4) Sacroiliac joint syndrome
5) Lumber compression fracture
6) Spinal Tumor
7) Lumber Disc Herniation
8) Infection
9) Lumber Spinal Stenosis
10) Ankylosing Spondylarthritis
11) Visceral Disease
12) Psychosocial factor
13) Others

Three groups were categorized: *S*, *Serious spinal disease* (5)-(11) *Diag*, *Diagnosable LBP* (1)-(4) but not a serious condition; *Not-Diag* (12)-(13), Not possible to make a definite diagnosis of LBP.

### Sensitivity and specificity of each test for the diagnosis of LBP

We next examined the sensitivity and specificity of each physical, neurological and radiographic finding and test in Fig 1 for the diagnosis of 1)-3) LBP in Table 2 because previous paper [2] reported the difficulty for the diagnosis with clear cause in these LBP classified as a non-specific LBP. To determine the overall diagnostic accuracy of each test, a 2 × 2 contingency table was created and the sensitivity and specificity along with their 95% confidence interval were calculated for each 1)-3) LBP group [11–13]. In the clinic, the rapid diagnosis of LBP is the next task following simple epidemiological investigation.

### Data analysis

Data input was through Access 2013 software (Microsoft, USA) and was transferred to the Statistical Package for Social Science (SPSS software, SPSS Inc., Chicago, IL) 13.0 for analysis.

## Results

### Differential diagnosis

**Fig 2:**
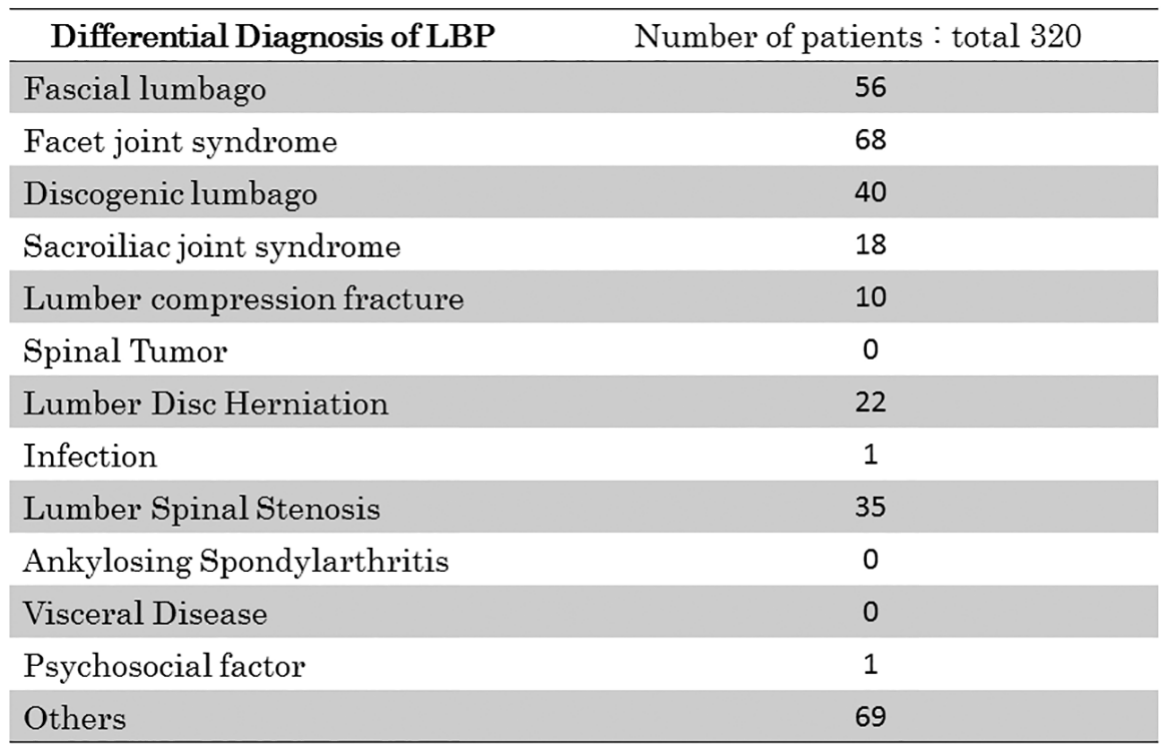
Summary of the Differential Diagnosis of LBP. Total number of LBP patients is 320.

**Fig 3:**
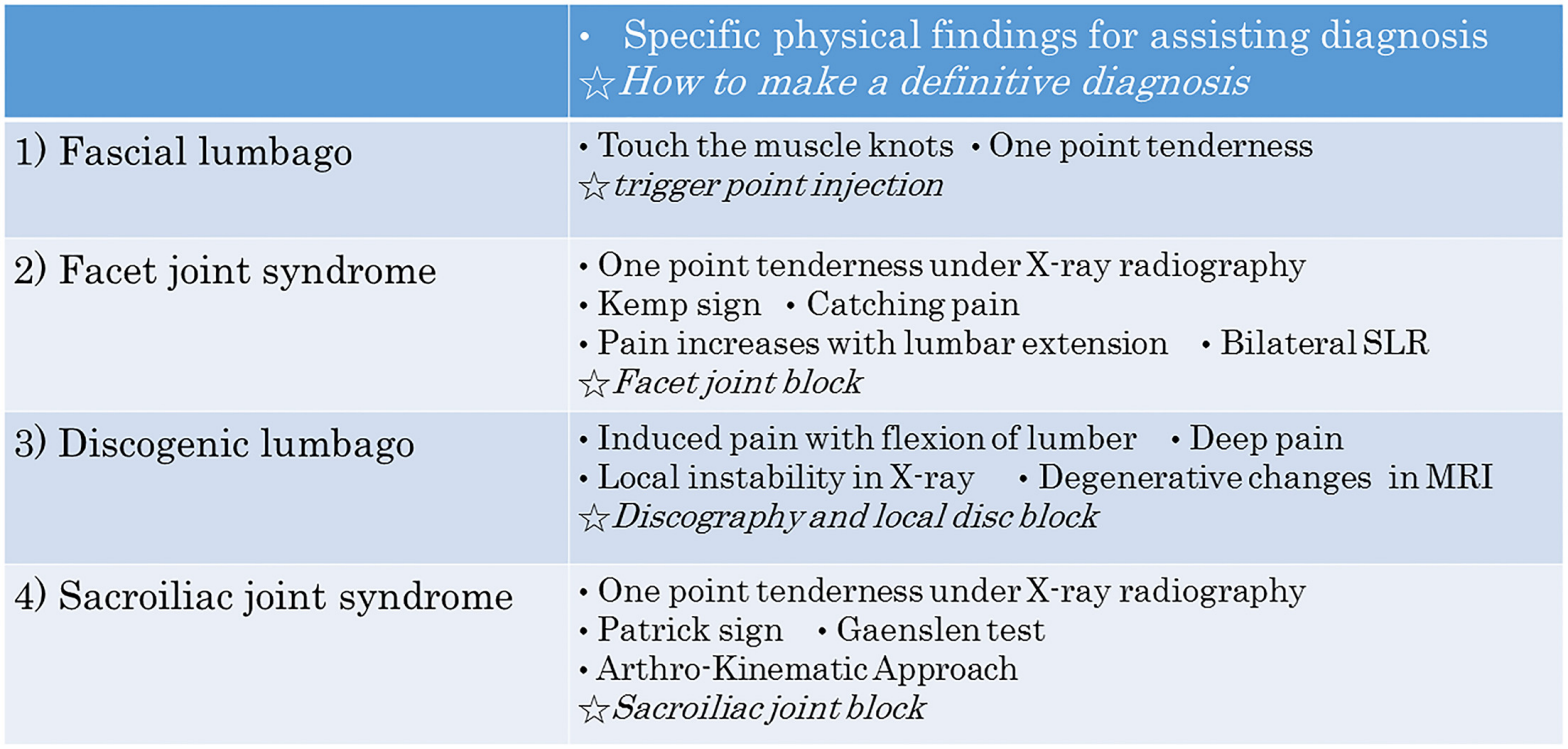
How to make a definitive diagnosis in each LBP. Specific physical findings for assisting diagnosis.

Fig 2 illustrates the differential diagnoses for LBP as defined in this study. Three groups were categorized: S, Serious spinal disease; Diag, Diagnosable LBP but not a serious condition; Not-Diag, Not possible to make a definite diagnosis of LBP. The S group represents the so-called ‘Specific LBP’ cases described in a previous paper [2], whereas the Diag and Not-Diag groups represent the previously described ‘Non-specific LBP’ [2]. Group S includes lumbar compression fracture (5), spinal tumor (6), lumbar disc herniation (7), infection (8), lumbar spinal stenosis (9), ankylosing spondylarthritis (10) and visceral disease (11). The Diag group includes fascial lumbago (1), facet joint syndrome (2), discogenic lumbago (3) and sacroiliac joint syndrome (4). The Not-Diag group is due to psychosocial (12) and other factors (13). The specific physical findings for (1) to (4) are described in Fig 3. The final diagnosis for types (1) to (4) LBP was performed by block injection of local anesthesia.

### Characteristics of LBP patients in Japan

**Fig 4:**
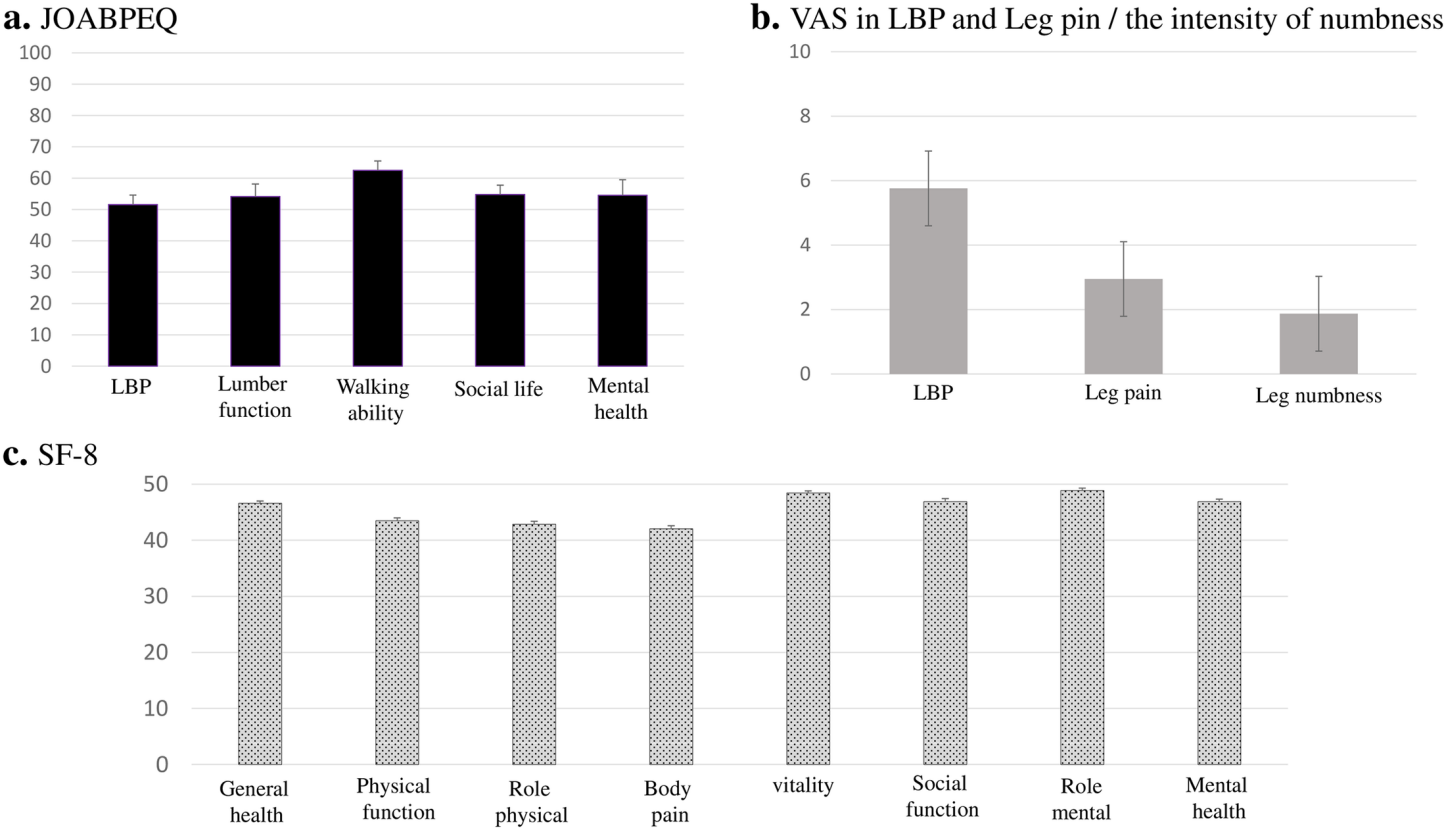
The background characteristics of patients in this study were assessed by JOABPEQ (a), VAS (b), and SF-8 (c).

Background characteristics of patients in this study (Table 3) were assessed by JOA score, VAS, JOABPEQ and SF-8 (Fig 4). The average JOA score was 19.7±0.27. The item scores for JOABPEQ were: LBP, 51.57±1.8; lumbar function, 54.1±1.7; walking ability, 62.5±1.8; social life, 54.8±1.3; mental health, 54.5±0.95 (Fig 4a). VAS scores were: LBP, 5.8±0.18; leg pain, 2.9±0.18; leg numbness, 1.9±0.16 (Fig 4b). Item scores for SF-8 were: general health, 46.6±0.40; physical function, 43.5±0.51; physical limitations, 42.8±0.53; body pain, 42.1±0.52; vitality, 48.4±0.37; social function, 46.9±0.53; emotional problems, 48.9±0.43; mental health, 46.9±0.43 (Fig 4c).

**Table 3:** Patient background.

Characteristic	
total	320
men	160
women	160
Ages	Avg. 55.7yrs (mean 20–85)
LBP duration	Avg. 433 days (mean 14–7369)
Duration of absence from work	Avg. 2.7 days (mean 0–730)

Background characteristics of patients, JOA score, VAS, JOABPEQ and SF-8 in group S, Diag and Not-Diag are shown in Table 4.

**Table 4:** Patient background in group S, Diag and Not Diag.

	GROUP S	GROUP DIAG.	GROUP NOT DIAG.
**Men**	37	87	36
**Women**	31	95	34
**Ages**	Avg. 65.2yrs (mean 20–83)	Avg. 53.8yrs (mean 20–85)	Avg. 54.8yrs (mean 20–85)
**LBP duration**	Avg. 660 days (mean 1–14600)	Avg. 387 days (mean 1–9125)	Avg. 300 days (mean 1–10950)
**Duration of absence from work**	Avg. 11.8 days (mean 0–730)	Avg. 4.1 days (mean 0–730)	Avg. 0.2 days (mean 0–1)
**JOA score**	17.2±4.71	20.6±4.51	19.4±4.7
**JOABPEQ**			
LBP	45.3±31.3	54.5±32.3	48.1±33.9
Lumbar function	54.4±31.1	54.5±31.1	48.7±31.2
Walking ability	47.8±31.7	66.4±30.1	62.2±33.8
Social life	45.8±22.2	58.4±22.2	51.6±25.4
Mental health	50.9±18.1	56.8±16.7	49.9±16.9
LBP (VAS)	5.7±2.3	5.7±2.5	6.1±2.5
Leg pain (VAS)	4.9±2.9	2.1±2.8	3.2±3.0
Leg numbness (1–10)	3.3±3.4	1.4±2.4	1.6±2.4
**SF-8**			
GENERAL HEALTH	44.6±7.5	47.7±7.2	45.6±6.3
PHYSICAL FUNCTION	40.4±10.7	44.9±8.3	42.6±8.6
PHYSICAL LIMITATIONS	39.3±10.3	44.5±8.9	41.8±9.4
BODY PAIN	40.0±9.7	43.2±9.2	41.1±9.1
VITALITY	46.8±7.0	49.4±6.4	47.4±6.1
SOCIAL FUNCTION	43.6±10.4	48.4±8.5	46.2±9.7
EMOTIONAL PLOBLEMS	47.4±8.1	50.5±6.5	45.9±8.6
** MENTAL HEALTH**	**43.9±8.7**	**48.5±6.9**	**45.5±7.6**

### Specific and non-specific LBP

In this study, group S comprised 21% (68) of cases, group Diag was 57% (182) and group Not-Diag was 22% (70). Since groups S and Diag were considered diagnosable LBP, the total Specific LBP cases was 78% (250). The proportion of non-specific LBP comprising the group Not-Diag was therefore just 22%.

### Sensitivity and specificity of each LBP test for diagnosis

**Fig 5:**
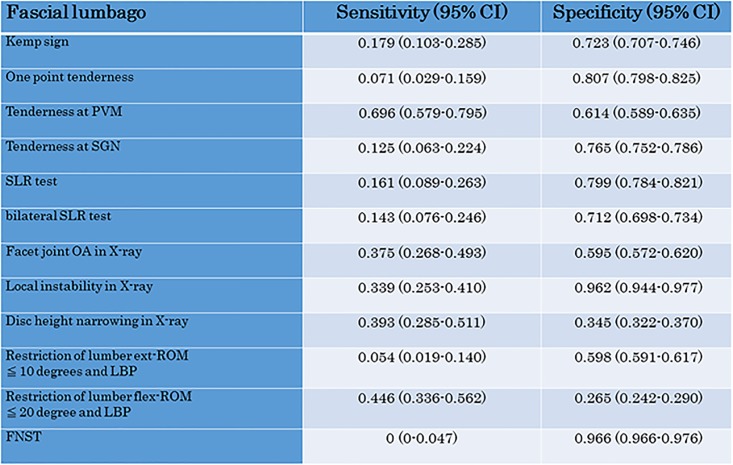
Sensitivity and specificity of the LBP test for Fascial lumbago.

**Fig 6:**
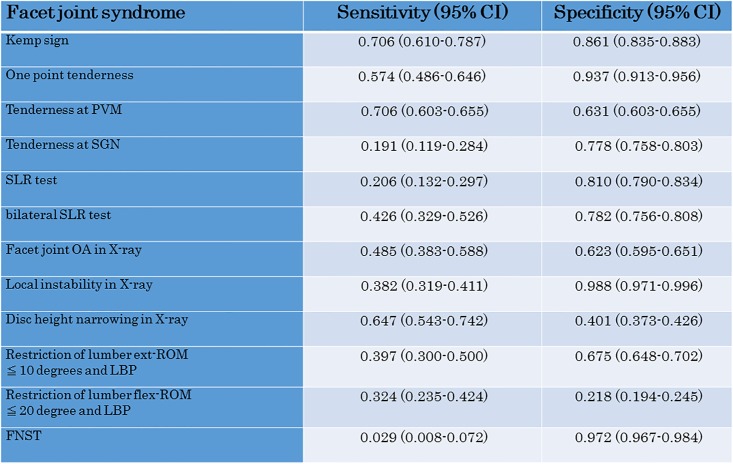
Sensitivity and specificity of the LBP test for Facet joint syndrome.

**Fig 7:**
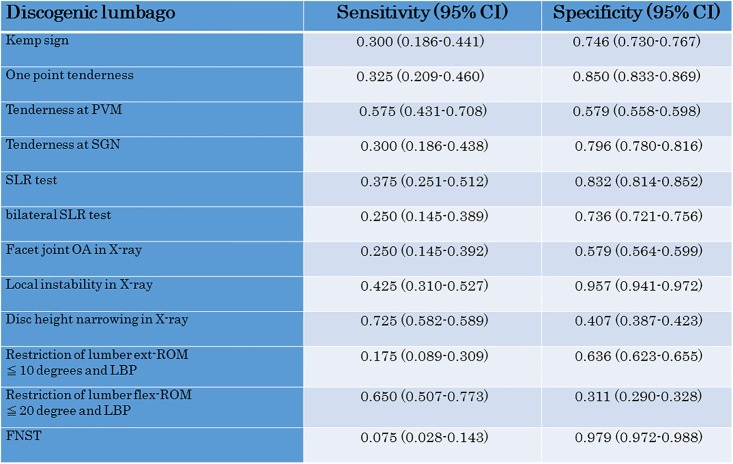
Sensitivity and specificity of the LBP test for Discogenic lumbago.

We also investigated the sensitivity and specificity of each test for fascial lumbago (1) (Fig 5), facet joint syndrome (2) (Fig 6) and discogenic lumbago (3) (Fig 7). With fascial lumbago, the sensitivity of tenderness at PVN was 0.696 and the specificity was 0.614. It was difficult to diagnose fascial lumbago based only on examination, with diagnosis requiring the exclusion of other causes of LBP. In facet joint syndrome, sensitivity of the kemp sign was 0.706 and specificity was 0.861, while sensitivity of one point tenderness was 0.574 and specificity was 0.937, and sensitivity of one point tenderness at PVM was 0.706 and specificity was 0.631. With discogenic lumbago, sensitivity of Disc height narrowing in X-ray was 0.725 and specificity was 0.407, while sensitivity of restriction of lumbar flex-ROM was 0.650 and specificity was 0.311.

## Discussion

LBP is one of the five most common health complaints in the Japanese general population [1]. Previous papers reported that approximately 80–85% of LBP cases in Western populations are classified as ‘non-specific’ [2,3] and do not experience severe disability [14]. In reality, however, a large proportion of LBP patients suffer mild to severe pain and experience a diminished QOL [15–17]. In addition some papers revealed that most of the LBP patients hadn’t had accurately diagnosis and effective treatment which lead to improve clinical outcome [18–20]. That’s the reasons why we focused on the diagnosis ratio in LBP in this study because the diagnosis could lead the effective therapy [18–20]. We therefore believe that specific therapies, not general treatment, can be performed for the most LBP patients whom we can diagnose what the cause of LBP clearly.

Achieving a clear and detailed diagnosis in non-specific LBP, it can lead to improved clinical outcomes because a clear diagnosis is essential for the early treatment of LBP cases [18–20]. The diagnosis and treatment of LBP in Japan is different to that reported previously in Western countries [2–3] due to differences in the health system [21]. In Japan, patients with LBP usually visit orthopedic clinics first and are therefore seen by orthopedists rather than by general practitioners.

The background characteristics of LBP patients in our study were evaluated by JOA score, JOABPEQ, VAS and the intensity of numbness. This revealed they mostly had mild LBP, with the major complaint being LBP without severe lower limb symptoms. The results showed their symptoms were mostly due to LBP, however the patients’ QOL scores were significantly affected.

The most important finding of this study was the differential diagnosis of LBP due to detailed examination carried out by an orthopedist. We were able to accurately diagnose 78% of LBP patients following detailed neurological, physical and radiological examinations. The incidence of group S, or the so-called ‘red flag LBP’, was almost the same (21%) as in previous reports [22–24]. However the incidence of the Diag group was 57%. Previous work suggested the detailed cause of LBP could not be identified in the majority of cases, or that it was not very important [2]. Our results show that 79% (57+22%) of LBP patients (Group Diag and Not Diag) who attend walk-in clinics in Japan do not have red flag LBP (group S), but nevertheless experience a reduced QOL and suffer from severe pain. Identification of a clear diagnosis of LBP for patients in group Diag should lead to improved treatment and better quality of care. A high diagnostic accuracy for LBP is very important in order to achieve excellent treatment results for patients with LBP.

In the differential diagnosis of LBP in this study, we did not include entrapment neuropathy of the superior cluneal nerve (SCN). Previous work reported that entrapment of the SCN should be considered as a cause of chronic LBP or leg pain [25]. Some of the patients classified as “others” in this study may therefore have had SCN disorder. To improve the diagnosis rate for LBP, it may therefore be necessary to consider SCN disorder.

We also investigated the sensitivity and specificity of each test for fascial lumbago, facet joint syndrome and discogenic lumbago, since these are the most common causes for non-specific LBP. Previous papers [2–3,14] reported they could not find clear causes of LBP in these types of LBP patients. Our data on the sensitivity and specificity of each test revealed there are some specific tests for each LBP, but that several examinations should be conducted to confirm the final diagnosis in LBP.

In conclusion, this study has shown that detailed examination, questioning and image diagnosis for LBP patients by Japanese orthopedists allows the identification of a high proportion of specific LBP cases. In reality the rate of non-specific LBP was only 22% in Japan. We were able to accurately diagnose 78% of LBP patients following detailed examinations. In addition, their symptoms were mostly due to LBP, however the patients’ QOL scores were significantly affected. Moreover, diagnosis of a clear cause of LBP allows treatment of the damaged lesion and can therefore lead to potentially better outcomes for more patients with LBP.
